# Deletion of DWORF does not affect cardiac function in aging and in PLN-R14del cardiomyopathy

**DOI:** 10.1152/ajpheart.00741.2023

**Published:** 2024-02-09

**Authors:** Nienke M. Stege, Vivian Oliveira Nunes Teixeira, Sietske N. Zijlstra, Anna M. Feringa, Rudolf A. de Boer, Herman H. W. Silljé

**Affiliations:** ^1^Department of Cardiology, University Medical Center Groningen, University of Groningen, Groningen, The Netherlands; ^2^Erasmus MC, Cardiovascular Institute, Thorax Center, Department of Cardiology, Rotterdam, The Netherlands

**Keywords:** cardiomyopathy, DWORF knockout, heart failure, p.Arg14del, phospholamban

## Abstract

The phospholamban (*PLN*) pathogenic gene variant p.Arg14del causes cardiomyopathy, which is characterized by perinuclear PLN protein clustering and can lead to severe heart failure (HF). Elevated expression of dwarf open reading frame (DWORF), a protein counteracting the function of PLN in the sarcoplasmic reticulum (SR), can delay disease progression in a PLN-R14del mouse model. Here, we evaluated whether deletion of DWORF (DWORF*^−/−^*) would have an opposite effect and accelerate age-dependent disease progression in wild-type (WT) mice and mice with a pathogenic PLN-R14del allele (R14*^Δ/+^*). We show that DWORF*^−/−^* mice maintained a normal left ventricular ejection fraction (LVEF) during aging and no difference with WT control mice could be observed up to 20 mo of age. R14*^Δ/+^* mice maintained a normal cardiac function until 12 mo of age, but at 18 mo of age, LVEF was significantly reduced as compared with WT mice. Absence of DWORF did neither accelerate the R14*^Δ/+^*-induced reduction in LVEF nor enhance the increases in gene expression of markers related to cardiac remodeling and fibrosis and did not exacerbate cardiac fibrosis caused by the R14*^Δ/+^* mutation. Together, these results demonstrate that absence of DWORF does not accelerate or exacerbate PLN-R14del cardiomyopathy in mice harboring the pathogenic R14del allele. In addition, our data indicate that DWORF appears to be dispensable for cardiac function during aging.

**NEW & NOTEWORTHY** Although DWORF overexpression significantly delayed heart failure development and strongly prolonged life span in PLN-R14del mice, the current study shows that deletion of DWORF does not accelerate or exacerbate PLN-R14del cardiomyopathy in mice harboring the pathogenic R14del allele. In addition, DWORF appears to be dispensable for cardiac function during aging. Changes in DWORF gene expression are therefore unlikely to contribute to the clinical heterogeneity observed in patients with PLN-R14del cardiomyopathy.

## INTRODUCTION

A large number of pathogenic gene variants have been identified to cause cardiomyopathies (1). The p.Arg14del mutation in the gene coding for phospholamban (PLN) can cause dilated cardiomyopathy (DCM) and arrhythmogenic cardiomyopathy (ACM), often resulting in severe heart failure (HF) (2, 3). There is a large clinical heterogeneity in age-related disease onset and severity of the symptoms among patients (4). Analysis of a limited number of patients’ hearts revealed that additional genetic variants might contribute to phenotypic diversity (5), yet the precise role and identity of these potential genes remain largely elusive.

PLN is a sarcoplasmic reticulum (SR) protein that can reduce the uptake of cytosolic calcium ions (Ca^2+^) into the SR via its modulating activity of the sarco/endoplasmic reticulum (S/ER) Ca^2+^-ATPase (SERCA) (6). Although PLN-R14del cardiomyopathy was originally described to be a disease driven by reduced SR-calcium uptake (3), more recent data show enhanced SR-calcium uptake in a PLN-R14del mouse model and in human induced pluripotent stem cells-derived cardiomyocytes (hiPSC-CM) (7, 8). Instead of altered calcium SR uptake, the formation of perinuclear PLN protein clusters that cause disorganization of the SR and subsequent cardiomyocyte death has recently emerged as the main mechanism for HF development in this cardiomyopathy (8–10).

We showed that overexpression of dwarf open reading frame (DWORF), a protein counteracting the function of PLN in the SR, delayed PLN-R14del cardiomyopathy and extended life span in a mouse model for this disease (8). Interestingly, the cardioprotective effect could not be attributed to the role of DWORF in calcium handling, but DWORF reduced the formation of harmful SR PLN protein clusters (8).

In several mouse models for HF and in patients with ischemic HF, a reduction in DWORF expression has been observed (11, 12). DWORF knockout (KO) mice are viable and showed a slight but significant decrease in the affinity of SERCA for calcium (11), which may contribute to disease development. However, the impact of DWORF deficiency on cardiac function in relation to aging has not been reported thus far. Given that HF is generally a disease of the elderly, it is conceivable that prolonged DWORF reduction may contribute to the development of HF. Interestingly, DWORF expression was also strongly decreased in homozygous PLN-R14del (R14*^Δ/Δ^*) mice, and the reduction was even initiated before cardiac impairment could be observed (8). This observed DWORF reduction coincided with S/ER malformation in R14*^Δ/Δ^* mice, which could indicate a causal relationship between DWORF expression levels and disease development. Since DWORF overexpression was able to counteract PLN-R14del disease development, we wondered whether the opposite, namely DWORF deficiency, could potentially aggravate disease progression. Since heterozygous PLN-R14del (R14*^Δ/+^*) mice develop disease slowly, with first signs of cardiac dysfunction at around 18 mo of age, these mice provide an excellent tool to investigate potential factors that could modulate disease progression (13). Here, we investigated whether DWORF deficiency could accelerate age-dependent disease development by deletion of DWORF in PLN-WT and PLN-R14*^Δ/+^* mice.

## METHODS

### Animals and Study Design

Animal studies were approved by the Central Committee of Animal Experiments (License No. AVD1050020199105) and the animal ethical committee of the University of Groningen (Permit No. IVD199105-01-008), conformed with the guidelines from Directive 2010/63/EU of the European Parliament, and reported following the Animal Research: Reporting of In Vivo Experiments (ARRIVE) guidelines (14). To evaluate the effect of DWORF deficiency on the development of PLN-R14del cardiomyopathy, DWORF*^−/−^* mice (11) were crossed with PLN-R14*^Δ/+^* mice (13) (R14*^Δ/+^* DWORF*^−/−^*). Genotyping was performed according to the methods described in the original articles. Mice were assigned to the experimental groups by their genotype, and both sexes were included. At 6, 12, and 18 mo of age, echocardiography and surface electrocardiography acquisition were performed in anesthetized mice (2.5% isoflurane mixed with oxygen) as described before (8) and were performed in a blinded manner. At 20 mo of age, mice were euthanized according to the procedure described before (8).

### Histology and Molecular Analysis

Masson’s trichrome staining and subsequent quantification of fibrosis were performed as described previously (8). Immunofluorescent (IF) staining for PLN (ab219626, Abcam, 1:1,000) with secondary antibody (A31572, Invitrogen, 1:100), together with fluorescein isothiocyanate (FITC)-conjugated wheat germ agglutinin (WGA; Sigma-Aldrich, 1:100) and 4′,6-diamidino-2-phenylindole (DAPI; Vector Laboratories), was performed to determine the abundance of PLN-containing protein clusters. PLN cluster-positive cardiomyocytes within a selected area of longitudinal cardiomyocytes were counted via a blinded procedure performed by an unbiased person. These data are presented as cluster-positive cells per mm^2^.

Gene expression of the genes listed in Table 1 (with corresponding primer sequences) was determined via quantitative polymerase chain reaction according to the procedure described before (8). The exported values of the genes of interest were normalized to the expression level of housekeeping gene *Rplp0* (36B4), and these ΔCt values are presented as fold change compared with the age-matched control group.

**Table 1.** Primer sequences used for qPCR

Gene	Forward Primer (5′–3′)	Reverse Primer (5′–3′)
*Rplp0* (36B4)	AAGCGCGTCCTGGCATTGTC	GCAGCCGCAAATGCAGATGG
*Nppa*	GCTTCCAGGCCATATTGGAG	GGTGGTCTAGCAGGTTCTTG
*Myh6*	AGCTCATGGCTACACTCTTC	GTGGGTGGTCTTCAGGTTTG
*Myh7*	GAGCATTCTCCTGCTGTTTC	GAGCCTTGGATTCTCAAACG
*Col1a1*	AGAGCATGACCGATGGATTC	CGCTGTTCTTGCAGTGATAG
*Timp1*	CAACGAGACCACCTTATACC	CATATCCACAGAGGCTTTCC

Western blot analysis and quantification were performed as described previously (8); however, this time detection was performed using an Amersham ImageQuant 800 Western blot imaging system (Cytiva). Primary anti-DWORF antibody [custom made; Nelson, Science 2016 (11)] 1:1,000 was used in combination with horseradish peroxide (HRP)-linked secondary goat anti-rabbit antibody (P044801, Agilent) 1:2,000, with Revert 700 Total Protein Stain (LI-COR Biosciences) as loading control. The calculated values are presented as fold change compared with WT control.

### Statistical Analyses

All data are presented as means ± SE. Because of small group sizes, the nonparametric Kruskal–Wallis test followed by Dunn’s post hoc test was performed for multigroup comparisons. All statistical analyses were performed using GraphPad Prism (Version 8.4.2, GraphPad Software). Comparisons made for all figures are WT vs. DWORF*^−/−^*, WT vs. R14*^Δ/+^*, DWORF*^−/−^* vs. R14*^Δ/+^*DWORF*^−/−^* and R14*^Δ/+^* vs. R14*^Δ/+^*DWORF*^−/−^*. *P* values <0.05 were considered statistically significant.

## RESULTS

### R14*^Δ/+^*-Induced Cardiac Impairment Is Neither Accelerated Nor Enhanced by DWORF Knockout

**Figure 1.**
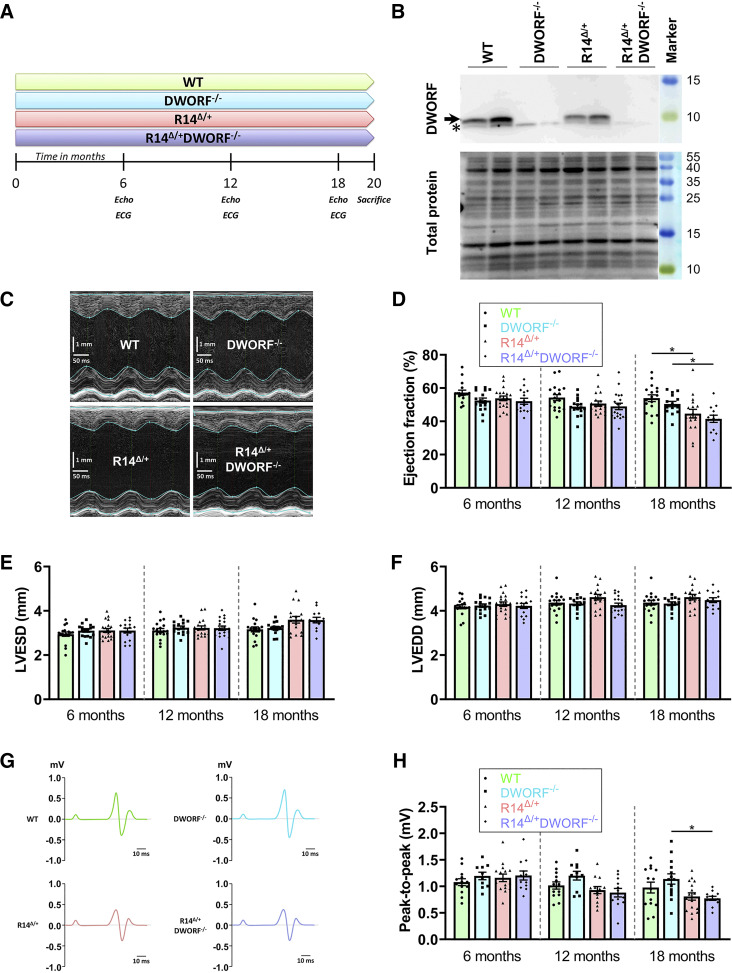
R14*^Δ/+^*-induced cardiac impairment is neither accelerated nor enhanced by DWORF knockout. *A*: overview of the experimental design. *B*: representative Western blot image of DWORF protein levels in the left ventricle (LV) of 20-mo-old WT, DWORF*^−/−^*, R14*^Δ/+^*, and R14*^Δ/+^*DWORF*^−/−^* mice (gels are cropped to improve clarity and conciseness of the figure, but space is retained above and below the relevant bands). Arrow indicates the DWORF-specific band, whereas asterisk below indicates a nonspecific band. *C*: representative serial M-mode echocardiographic images of the LV at 18 mo of age (vertical scale bar for *y*-axis, 1 mm and horizontal scale bar for *x*-axis, 50 ms) with quantification of the ejection fraction (*D*), LV end-systolic diameter (LVESD; *E*), and LV end-diastolic diameter (LVEDD; *F*) at 6, 12, and 18 mo of age. *G*: averaged ECG traces, where the average from 1-min ECG recordings of multiple 18-mo-old mice was used. The *x*-axis per ECG complex has a duration of 90 ms (scale bar, 10 ms). *H*: quantification of the ECG R-to-S peak-to-peak amplitude with wild-type in green, DWORF*^−/−^* mice in light blue, R14*^Δ/+^* mice in red, and R14*^Δ/+^*DWORF*^−/−^* mice in purple. Significance was examined by Kruskal–Wallis with Dunn’s multiple comparisons test. *P < 0.05. DWORF, dwarf open reading frame; R14*^Δ/+^*, PLN-R14del allele; WT, wild type.

It was shown previously that cardiac dysfunction was present at 18 mo of age in PLN-R14*^Δ/+^* mice (13). Since a potential acceleration of phenotype could be expected in the R14*^Δ/+^*DWORF*^−/−^* mice, echo- and electrocardiography were performed at 6, 12, and 18 mo of age in this study, as outlined in Fig. 1*A*. Finally, mice were terminated at 20 mo of age for histological and molecular analyses. Western blot analysis confirmed cardiac knockout of DWORF in DWORF*^−/−^* and R14*^Δ/+^*DWORF*^−/−^* mice (Fig. 1*B*). Moreover, quantification showed that DWORF protein level was ∼50% lower in the R14*^Δ/+^* mice as compared with WT mice (0.54 ± 0.18 fold change for R14*^Δ/+^* compared with 1.00 ± 0.32 fold change for WT, *P* < 0.05 and *n* = 8 each). This is in line with previous observations showing a strong reduction in homozygous R14*^Δ/Δ^* mice (8).

Using echocardiography, cardiac function was monitored over time. Up to 12 mo of age, left ventricular ejection fraction (LVEF) was normal in all mouse strains. At 18 mo of age, LVEF of PLN-R14*^Δ/+^* mice was significantly decreased compared with wild-type (WT) mice, whereas left ventricular end-systolic diameter (LVESD) and left ventricular end-diastolic diameter (LVEDD) were equal between both genotypes (Fig. 1, *C–F*). These data are in line with initial publication of the PLN-R14*^Δ/+^* mouse model (13). DWORF knockout did not have an effect on cardiac function in a WT background, and LVEF remained normal, even at 18 mo of age. A significant reduction in cardiac function was observed in R14*^Δ/+^*DWORF*^−/−^* mice at 18 mo of age, but to a similar extent as in R14*^Δ/+^* mice (Fig. 1, *C*–*F*). Heart rate was comparable between the different genotypes (Table 2). Diminished ECG potentials are often present in PLN-R14del cardiomyopathy, and therefore the ECG potentials were determined and the peak-to-peak amplitude (R-to-S) quantified at all time points. Although some trend of lower amplitude was observed for the R14*^Δ/+^* strains, this was only significant for the R14*^Δ/+^*DWORF*^−/−^* at 18 mo of age, and no significant difference between R14*^Δ/+^* and R14*^Δ/+^*DWORF*^−/−^* could be observed (Fig. 1, *G* and *H*).

**Table 2.** No differences in heart rate between the genotypes

	Heart Rate, Beats/Min		
Genotype	6 mo	12 mo	18 mo
WT	445.6 ± 8.8	420.7 ± 7.4	417.4 ± 8.2
DWORF*^−/−^*	421.2 ± 14.4	407.1 ± 5.9	406.8 ± 3.6
R14*^Δ/+^*	431.8 ± 6.4	398.5 ± 6.8	417.7 ± 6.7
R14*^Δ/+^*DWORF*^−/−^*	428.4 ± 8.6	404.3 ± 6.6	419.9 ± 6.4

Values are presented as means ± SE. Heart rate measurements were performed in anesthetized mice (2.5% isoflurane mixed with oxygen). Significance was examined by Kruskal–Wallis with Dunn’s multiple comparisons test; however, there were no significant differences between the genotypes. WT, wild type; DWORF, dwarf open reading frame; R14*^Δ/+^*, PLN-R14del allele.

### R14*^Δ/+^*-Induced Cardiac Remodeling Is Not Exacerbated by DWORF Knockout

**Figure 2.**
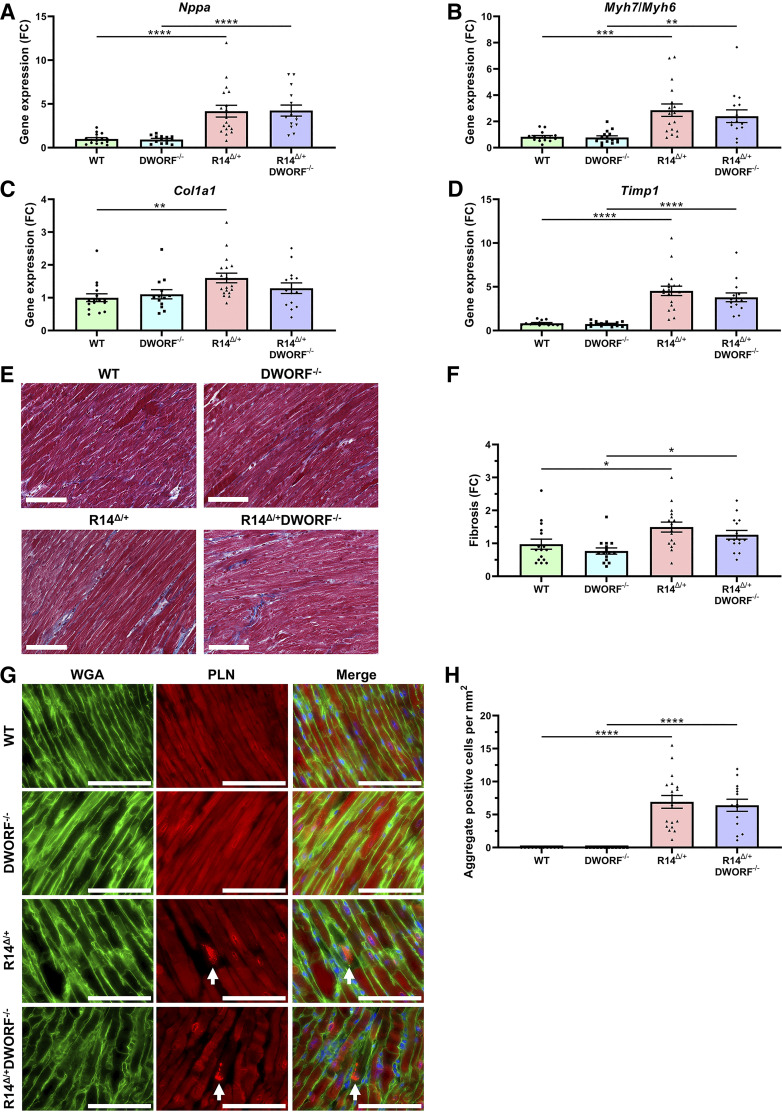
R14*^Δ/+^*-induced cardiac remodeling is not exacerbated by DWORF knockout. Relative left ventricular (LV) gene expression of the HF marker *Nppa* (*A*), the *Myh7*:*Myh6* ratio (*B*), and fibrotic markers *Col1a1* (*C*) and *Timp1* (*D*) at 20 mo of age, measured by qPCR and displayed as a fold change (FC) difference to wild-type (WT) control. *E*: representative Masson’s trichrome-stained cardiac tissue sections of 20-mo-old WT, DWORF*^−/−^*, R14*^Δ/+^*, and R14*^Δ/+^*DWORF*^−/−^* mice (scale bar, *bottom left*, 100 µm). *F*: quantification of cardiac fibrosis displayed as a fold change (FC) difference to WT control (*n* = 15–18/group). *G*: representative images of WGA staining (green) showing cell boundaries, IF staining of PLN (red), and a merge of these channels together with DAPI staining the nuclei (blue) in 20-mo-old LV tissue sections (scale bar, *bottom right*, 100 µm). Examples of PLN clusters are indicated by white arrowheads. *H*: PLN cluster-positive cardiomyocyte count per mm^2^ (*n* = 15–18/group). Significance was examined by Kruskal–Wallis with Dunn’s multiple comparisons test. *P < 0.05; **P < 0.01; ***P < 0.001; and ****P < 0.0001. PLN, phospholamban; WGA, wheat germ agglutinin.

Consistent with the reduced EF in R14*^Δ/+^* and R14*^Δ/+^*DWORF*^−/−^* mice at 18 mo of age, gene expression of the HF marker *Nppa* [atrial natriuretic peptide (ANP)] and the ratio myosin heavy chain 7, encoding β-MHC, and myosin heavy chain 6, encoding α-MHC (*Myh7*/*Myh6*) were increased in these groups compared with the expression in aged WT mice (Fig. 2, *A* and *B*). DWORF knockout had no effect on the expression of these genes in the WT-PLN background and did not contribute to the altered expression in the PLN-R14*^Δ/+^* background. Gene expression of the fibrotic markers collagen type Iα1 chain (*Col1a1*) and tissue inhibitor of metalloproteinase 1 (*Timp1*) was elevated in aged PLN-R14*^Δ/+^* mice (Fig. 2, *C* and *D*). DWORF knockout had no effect on the expression level of these fibrotic genes in both WT and R14*^Δ/+^* mice. To corroborate this, Masson’s trichrome staining was performed on histological heart sections and showed a significant increase in cardiac fibrosis in aged R14*^Δ/+^* and R14*^Δ/+^*DWORF*^−/−^* mice, without a difference between these groups (Fig. 2, *E* and *F*). Since abnormal PLN cluster formation is associated with disease progression, PLN cluster-positive cardiomyocytes were quantified in mice at 20 mo of age. PLN cluster-positive cardiomyocytes were absent in WT and DWORF*^−/−^* mice (Fig. 2, *G* and *H*). However, PLN cluster-positive cardiomyocytes were significantly elevated in R14*^Δ/+^* and R14*^Δ/+^*DWORF*^−/−^* mice, although the increase was similar between these two groups. Together, the molecular and histological analysis revealed that DWORF knockout does not cause age-related cardiac dysfunction and does not enhance or accelerate R14*^Δ/+^*-induced cardiac remodeling.

## DISCUSSION

In this study, we demonstrate that deletion of DWORF does not cause age-dependent cardiac dysfunction and does not enhance or accelerate PLN-R14del cardiomyopathy in a heterozygous mouse model of this disease. These findings indicate that DWORF is dispensable for cardiac function under these conditions.

There exists a continuum of cardiovascular structural and functional alterations during “normal” aging in both mice and humans. Shared cardiac changes in these species include an increase in LV wall thickness (hypertrophy), a decline in LV early diastolic filling (indicative of diastolic dysfunction), and an increase in cardiac fibrosis (15). Also at the molecular level, similar pathway changes are observed during aging. Although mice are more resilient for cardiac arrhythmias, the murine model overall closely recapitulates the age-related cardiac changes observed in humans. Consequently, mice serve as a valuable tool to evaluate the relevance of specific proteins during cardiac aging. Both in the aging heart and in HF, SERCA2a/PLN ratios and/or activity have been shown to decrease in humans and mice, which is believed to contribute to detrimental changes in calcium handling (16). Since DWORF counteracts the inhibitory PLN function, we investigated the effect of DWORF deletion in aged mice. Surprisingly, we did not observe cardiac dysfunction in DWORF KO mice with age, suggesting that decreased DWORF levels do not play a role in age-related disease development. This may explain why pathogenic mutations in humans have not yet been identified in this gene and no HF-related DWORF polymorphisms have been described so far. We like to note that caution should be taken in directly translating the absence of a mouse DWORF KO cardiac phenotype to humans since species-specific phenotypes can exist (17) and the sequence of mouse and human DWORF slightly differs from each other (11).

For PLN-R14del cardiomyopathy, the striking resemblance of human disease recapitulated by the mouse model has been extensively described before (13). Importantly, we previously showed that DWORF levels are exceptionally low in a homozygous mouse model for this disease, compared with mice with myocardial ischemia reperfusion (I/R) injury and mice that underwent myocardial infarction (MI) (8). We now also confirmed lower DWORF levels in aged heterozygous PLN-R14del mice as compared with WT control. A reduction in DWORF expression has also been observed in patients with HF (11), but whether this contributes to HF development is unknown. Together with the observation that DWORF overexpression delays disease development in homozygous PLN-R14del mice (8), these findings prompted us to investigate whether a reduction in DWORF could accelerate or exacerbate PLN-R14del cardiomyopathy. So far, factors that promote disease onset and severity in PLN-R14del carriers have remained unidentified. For example, frequent exercise is associated with an earlier onset of ACM in desmosomal gene variant carriers but does not influence PLN-R14del cardiomyopathy development (18). Hence the current study evaluated the effect of complete absence (DWORF*^−/−^*) on PLN-R14del disease development. Importantly, our data show that changing normal physiological DWORF expression to its complete absence did not affect disease progression in R14*^Δ/+^* mice. It is therefore unlikely that differences in DWORF expression can contribute to the clinical heterogeneity observed in patients. As a change from the physiological level to the absence of DWORF does not affect PLN-R14del disease development, while supraphysiological DWORF expression clearly improves cardiac function (8), these data indicate that the protective effect of high DWORF levels is likely achieved through a competition effect within the S/ER. This competition may involve not only SERCA but also other interacting proteins and requires more elaborate future investigations, which could provide intriguing new leads for the cardioprotective mechanisms of DWORF in PLN-R14del cardiomyopathy.

Although absence of DWORF does not exacerbate PLN-R14del cardiomyopathy in mice, it is of importance to note that PLN-R14del cardiomyopathy has a distinct disease mechanism compared with other HF etiologies, including abnormal S/ER clustering (8) which, to our knowledge, has not been observed in other types of HF. In specific, DWORF overexpression attenuated PLN-R14del cardiomyopathy in mice via inhibition of abnormal PLN S/ER-clustering, since DWORF could not further enhance the already accelerated calcium reuptake in PLN-R14del mice (8). However, DWORF overexpression attenuated disease via stimulation of SERCA activity in other mouse HF models (12, 19). The enhanced calcium reuptake in PLN-R14del could therefore compensate for calcium effects of DWORF deficiency, but this compensatory effect is absent in other forms of HF. Therefore, we cannot rule out the possibility that reduced DWORF levels may contribute to other types of HF.

Finally, we like to note that although both sexes were included, this study was not designed and powered to detect differences between the sexes. Therefore, the absence of significant differences does not necessarily exclude the presence of potential differences. A recent human cohort study showed that low-voltage ECGs had more prognostic value in males, suggesting that male/female differences can be present in PLN-R14del cardiomyopathy (20).

Together, we demonstrate that deletion of DWORF does not cause cardiac dysfunction in aging mice and does not accelerate or exacerbate PLN-R14del cardiomyopathy in R14*^Δ/+^* mice. However, because of the unique PLN-R14del cardiomyopathy disease mechanism, we cannot exclude detrimental effects of low DWORF levels in other types of cardiac disease.

## DATA AVAILABILITY

Data supporting this study are available upon request.

## GRANTS

This work was supported by The Netherlands Heart Foundation (CVON PREDICT2) Grant 2018-30, the Leducq Foundation (Cure PhosphoLambaN-induced Cardiomyopathy, Cure-PLaN), and grants from The Netherlands Heart Institute and PLN foundation.

## DISCLOSURES

The UMCG, which employs several of the authors, has received research grants and/or fees from AstraZeneca, Abbott, Boehringer Ingelheim, Cardior Pharmaceuticals GmbH, Ionis Pharmaceuticals, Inc., Novo Nordisk, and Roche. Rudolf A. de Boer received speaker fees from Abbott, AstraZeneca, Bayer, Novartis, and Roche. None of the other authors has any conflicts of interest, financial or otherwise, to disclose.

## AUTHOR CONTRIBUTIONS

N.M.S. and H.H.W.S. conceived and designed research; N.M.S., V.O.N.T., S.N.Z., and A.M.F. performed experiments; N.M.S. analyzed data; N.M.S. and H.H.W.S. interpreted results of experiments; N.M.S. prepared figures; N.M.S. and H.H.W.S. drafted manuscript; N.M.S., R.A.d.B., and H.H.W.S. edited and revised manuscript; N.M.S., V.O.N.T., S.N.Z., A.M.F., R.A.d.B., and H.H.W.S. approved final version of manuscript.
